# Miniaturized Active-Frequency Selective Surfaces for Low-Power Internet of Things Devices

**DOI:** 10.3390/mi15060736

**Published:** 2024-05-31

**Authors:** Liang Zhang, Haobin Yang, Yan Wang, Shaoqing Zhang, Tongyu Ding

**Affiliations:** 1 School of Ocean Information Engineering, Jimei University, Xiamen 361021, China; liangzhang@jmu.edu.cn (L.Z.); h.yang@jmu.edu.cn (H.Y.); 2 Shenyang Aircraft Design and Research Institute, Shenyang 110035, China; sy601wangyan@163.com

**Keywords:** active-frequency selective surface, mode conversion, low-power communication, microwave, IoT, backscatter

## Abstract

With the proliferation of smart devices, the Internet of Things (IoT) is rapidly expanding. This study proposes a miniaturized controllable metamaterial with low control voltage for achieving low-power and compact designs in IoT node devices. Operating at a target frequency of 2.4 GHz, the proposed metamaterial requires only a 3.3 V control voltage and occupies approximately one-third of the wavelength in size. Experimental validation demonstrates its excellent reflective control performance, positioning it as an ideal choice for low-power IoT systems, particularly in the context of miniaturized and low-power IoT node applications.

## 1. Introduction

With the increasing prevalence of smart devices, the Internet of Things (IoT) is experiencing rapid growth [1]. IoT power-saving devices typically require low-power and compact designs. Controllable metamaterials loaded with varactor diodes, serving as nodes in backscatter communication systems [2], offer significant flexibility advantages. However, these controllable metamaterials usually require high control voltages, which makes them incompatible with the low-power requirements of IoT applications. Although the surface itself consumes very little power, the associated high-voltage control circuits are challenging to realize in a low-power, cost-effective manner. Additionally, the controllable metamaterials reported to date are often large in size, making them unsuitable for miniaturized power-saving devices.

Recent research endeavors aim to elucidate the model of low-power backscatter Internet of Things (IoT) communication systems [3] based on reconfigurable intelligent surfaces [4,5] within a general or specific system framework. In this communication model, low-power reconfigurable intelligent surfaces play a vital role [6,7]. According to the analysis of demands in IoT scenarios [8,9], reconfigurable surfaces such as active-frequency selective surfaces (AFSSs) should possess characteristics such as miniaturization, low power consumption, and high modulation efficiency when designed [10,11,12]. Therefore, the design of AFSS structures is undertaken.

As is known, AFSSs are typically implemented by integrating diodes or varactors [13], with diode-mounted AFSSs capable of converting between transmission and reflection modes by adjusting the diodes to ’on’ and ’off’ states. For example, in [14], an AFSS achieving full reflection and 99% transmission at 3.5 GHz was proposed, where the diodes are forward biased and reverse biased, respectively. These AFSSs are compatible with the WiFi frequency band commonly used in IoT scenarios. However, they demand precise control over the diodes’ mode switching between forward and reverse biasing, which poses challenges in circuit design. In order to simplify circuit complexity and enhance tuning flexibility, AFSSs integrated with varactors emerge as a promising alternative. For instance, in [15], multifunctional and miniaturized flexible AFSSs were proposed, achieving frequency selection within the range of 8–12 GHz under bias voltages ranging from 0 to 55 V. Nevertheless, the structures remain complex, and the use of bias networks leads to higher power consumption, making them unsuitable for low-power IoT scenarios.

Considering the demands for miniaturization and low power consumption in IoT scenarios [16], we propose a hypothesis to design an AFSS compatible with the WiFi frequency band and requiring only small voltage operation. In this paper, this hypothesis provides a framework for designing AFSSs tailored for low-power IoT devices. In [17], a serrated structure AFSS was proposed, offering a wide adjustable range from 935 MHz to 2.405 GHz under bias voltages from 0 to 30 V. This concept paves the way for the following design exploration: Can we achieve frequency tuning around 2.4 GHz with a minimal bias voltage, for example, 3.3 V? According to the data provided in [17], varying the bias voltage from 0 to 3.3 V results in the frequency range being tuned within 935 MHz to 1.35 GHz. Utilizing the resonance frequency formula, it increase the operating frequency to 2.4 GHz by reducing the inductance value, which can be achieved by downsizing the AFSS unit. Hence, we assert that this serrated structure holds significant potential.

This paper introduces a serrated structure, presenting the design and fabrication of a miniaturized AFSS tailored for low-power IoT applications. During the AFSS design process, analysis and optimization of the equivalent circuit diagram of the serrated structure were conducted. The AFSS prototype operates at 2.4 GHz, highly relevant to our targeted IoT environment, with a voltage range of 0–3.3 V. Additionally, it boasts compact dimensions, measuring only 5.5×5.6 mm${}^{2}$. The first section elucidates the practical scenarios and requirements of IoT node devices, establishing objectives tailored for low-power IoT devices. The second section outlines the methodology adopted in this research, summarizing the design principles and theoretical analysis of the AFSS. The third section validates the designed AFSS through simulation, while the fourth section introduces a direct testing method and provides corresponding results. Finally, the fifth section provides a comprehensive summary of the entire paper.

## 2. Design and Analysis

The resonant frequency of AFSSs is often described using LC resonant equivalent circuits and can generally be estimated using the resonant frequency [18]. For AFSSs utilizing varactors, the resonant frequency is typically represented by (1), where $C_{T}$ denotes the adjustable capacitance of the variable, ΔC is the parasitic capacitance generated by the metal strip, $L_{T}$ is the conductor inductance generated by the metal strip, and ΔL represents the mutual inductance between units. In the central frequency formula, it is observed that when the adjustable range of the variable is fixed, the overall frequency tuning range can be extended by increasing the mutual inductance of the AFSS unit.

(1) $$f_{0}=\frac{1}{2\pi \sqrt{\left(L_{T}+\Delta L\right)\left(C_{T}+\Delta C\right)}}$$

**Figure 1:**
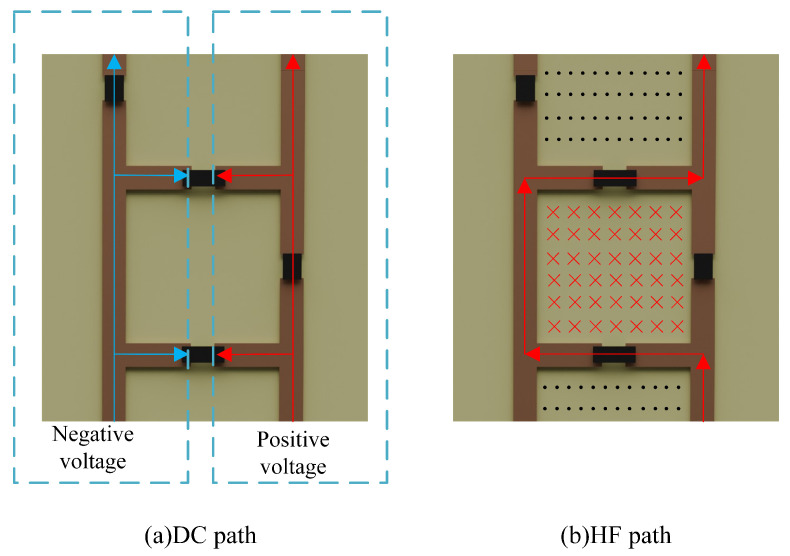
Structure of AFSS unit and analysis.

This paper introduces a serrated structure design. In this serrated structure, frequency adjustability is achieved using varactor diodes (specifically, the varactor BB857 varactor diode produced by Infineon). The BB857 features a wide adjustable capacitance range, $C_{T}$, from 0.54 to 6.6 pF under a bias voltage range of 0 to 30 V, with a low series resistance, $r_{s}$, of 1.5 Ω and a series inductance, $L_{S}$, of 0.6 nH. The varactor diode is reverse biased, with a maximum current of 200 nA at $V_{R}$ = 30 V and a minimum resistance of 150 MΩ. As shown in Figure 1, two 10 kΩ resistors are added to the structure to separate DC and high-frequency (HF) signals into different paths.

As illustrated in Figure 1a, by applying bias voltage through the bottom, the DC voltage can be transferred to the top of the column via the resistor to provide reverse biasing for the varactor. Notably, we focus on modulation capabilities with bias voltages ranging from 0 to 3.3 V. According to the BB857 datasheet, we found that the adjustment range of this varactor diodes is from 3.5 to 6.6 pF. Compared to the internal resistance of the varactor (150 MΩ), the resistance value of the resistor (10 kΩ) is relatively small; thus, the voltage drop across the resistor can be neglected. Additionally, the resistor inhibits the transmission of HF signals along the path of HF currents. This design utilizes the resistor as the impedance for HF signals, minimizing the bias’s impact on HF signals. As shown in Figure 1b, due to the relatively small resistance value of the resistor, HF signals form a zigzag surface current flow through the varactors. This provides insights for designing miniaturized AFSSs with control over a smaller voltage range.

**Figure 2:**
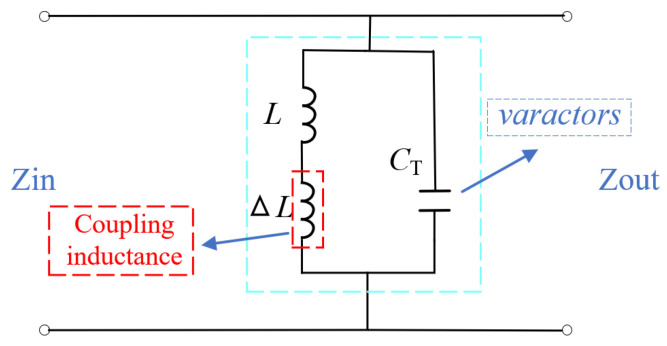
Simplified equivalent circuit model of the proposed AFSS.

**Figure 3:**
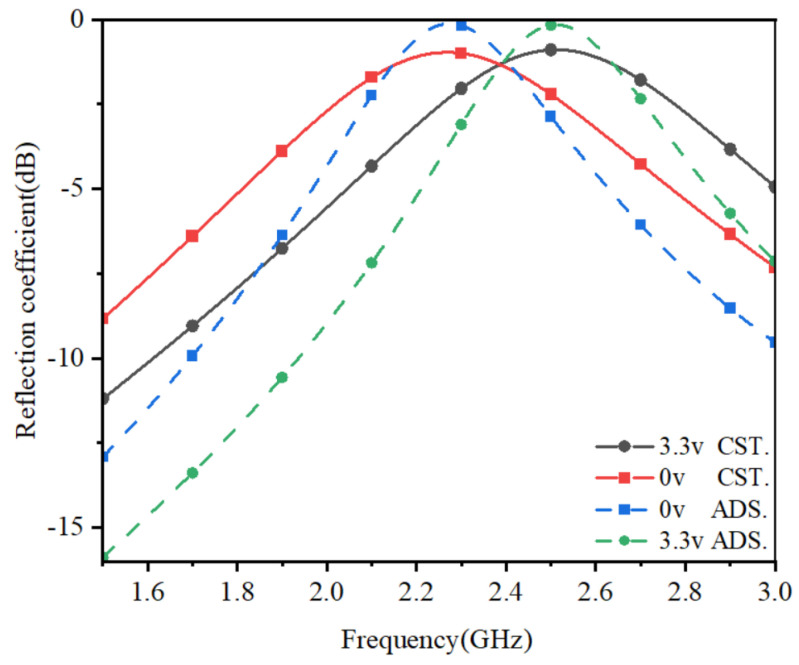
Simulation of continuously tunable ability. The capacitance of the varactors varies from 6.6 to 3pF when they are biased.

The HF equivalent circuit diagram of AFSS units with serrated structure is illustrated in Figure 2. Here, *L* represents the intrinsic inductance of the structure, $C_{T}$ denotes the capacitance value of the varactor controlled by bias voltage, and ΔL signifies the mutual inductance generated by the series connection of units. Before introducing the serrated structure, the series connection of multiple units did not effectively increase ΔL. To achieve miniaturization of AFSS units, we estimated the theoretical minimum inductance of the unit. In advanced design system (ADS 2020) simulations, we varied the value of $C_{T}$ corresponding to the capacitance value for the voltage control range, as shown in Figure 3. When the number of units increases to six, effective reflection and transmission selection can be achieved within the 2.4 GHz range.

## 3. Simulation Validation and Analysis

**Figure 4:**
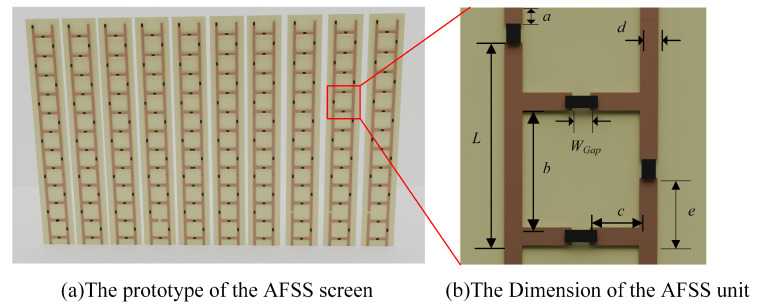
Conceptual configuration of the proposed AFSS with functional switching between absorption and transmission. (*L* = 4.2 mm, a = 1 mm, b = 2.4 mm, c = 1.5 mm, d = 0.4 mm, e = 1.4 mm, $G_{\mathrm{ap}}$ = 0.4 mm).

Based on the simulation results of the equivalent circuit, this paper presents the design of miniaturized AFSSs for IoT low-power devices. The conductor inductance values of the units are correlated with the size of the structure. After analyzing the equivalent circuit diagrams, this paper designed the unit structure and dimensions, as shown in Figure 4b. The prototype features a single-layer structure, with the substrate made of FR-4, and the unit size measures only 5.5 × 5.6 mm${}^{2}$.

The proposed zigzag AFSS structure effectively enhances the mutual inductance between AFSS units, thereby reducing the size and number of column units. To validate the HF equivalent circuit model simulation results, as shown in Figure 4a, it is assumed that the AFSS consists of six units in series on a plane consistent with the AFSS simulation, and the reflection coefficient of the AFSS is simulated using Computer Simulation Technology(CST 2022)’s frequency domain solver. Two Floquet ports are used, one in front of the AFSS and the other behind it. In CST, lumped components can be represented by RLC series, RLC parallel, or diode models. Based on the varactor diode Simulation Program with Integrated Circuit Emphasis (SPICE) model, it is expected to implement a small-signal HF equivalent circuit in RLC series form. Therefore, in the CST simulation validation we adopted the RLC series model, where the series resistance ($r_{s}$) is 1.5 Ω, the series inductance ($L_{S}$) is 0.6 nH, and the continuously adjustable capacitance ($C_{T}$) ranges from 3 to 6.6 pF. As shown in Figure 3, the adjustable range is found to be from 2.26 to 2.5 GHz in the CST simulation, with dimensions only one-third of the wavelength. Furthermore, the structure achieves a low insertion loss of less than 3 dB within the passband. The CST simulation results are consistent with those of ADS.

During operation, the varactor diodes in the AFSS are biased in the reverse bias state, and the operating current of the AFSS is approximately in the nanoampere level. Due to the limitations of the equipment precision, the exact operating current of the AFSS in its working state cannot be obtained. However, the bias voltage of the AFSS is determined to be 3.3 V. Additionally, the varactor diodes used in this study are Infineon’s BB857, whose datasheet indicates a reverse bias resistance of 5500 MΩ at room temperature based on the V-I curve for 3.3 V. Therefore, based on the voltage and resistance, the power consumption of the AFSS during operation can be estimated. In this study, the AFSS consists of ten metamaterial strip topologies. Using the basic circuit power definition under the aforementioned conditions, the power consumption of the AFSS is derived to be 237.6 nW. Considering the potential variations in resistance and operating conditions, the estimated power consumption of the AFSS ranges from nanowatts (nW) to microwatts (μW).

## 4. Experiment Verification

### 4.1. Fabrication and Experiment

**Figure 5:**
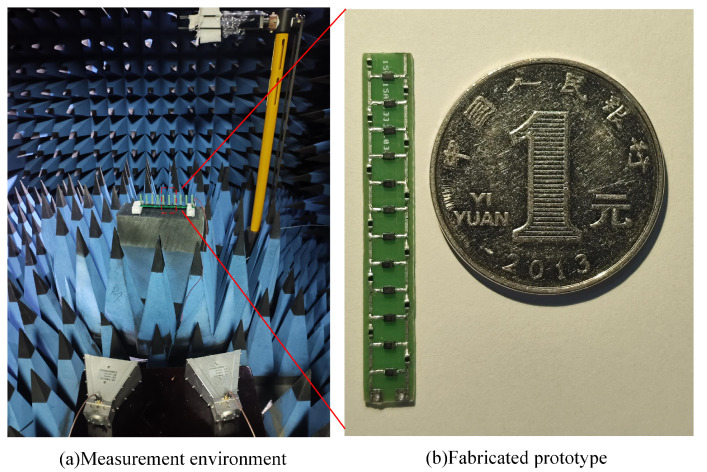
Fabricated AFSS and measurement setup.

This study proposes a miniaturized AFSS antenna system for IoT micro-devices. We fabricated an AFSS screen with ten columns, each column containing six units, resulting in a total length of 37 mm. As shown in Figure 5b, the overall dimensions are only 5.5×37 mm${}^{2}$.

Ref. [19] investigates the propagation process within communication systems. It emphasizes that the performance of the modulation screen in the channel link depends on its Radar Cross Section (RCS). However, due to the small overall size of the surface designed in this paper, accurate RCS values cannot be obtained in the existing experimental environment. Therefore, we propose a more efficient and practical method to measure the modulation capability of the screen. The equation for RCS variation, denoted as $\Delta \sigma_{\mathrm{RCS}}$, is presented as follows:

(2) $$\begin{array}{c} \Delta \sigma_{RCS}=\frac{\lambda^{2}G_{t}^{2}}{4\pi }{\left|\Gamma_{A}-\Gamma_{B}\right|}^{2}. \end{array}$$

Here, λ symbolizes the wavelength of the incident signal, $G_{t}$ represents the gain of the transmitting antenna, and $\Gamma_{A}$ and $\Gamma_{B}$ denote the reflection coefficients corresponding to two distinct states of the modulation screen. The squared absolute difference between $\Gamma_{A}$ and $\Gamma_{B}$ serves as a quantitative measure of the modulation-induced alteration in reflected signal strength, thereby highlighting the screen’s dynamic capability to adjust its reflective characteristics.

In the non-guided calibration of a vector network analyzer, the through mode is a calibration method used to eliminate the influence of circuits near the measurement ports on the measurement results. In the through mode, the measurement ports are connected to the network analyzer and are connected to the load or reference plane via a series of standardized transmission lines. This ensures that the signal path near the measurement ports remains unchanged during the measurement, thereby improving measurement accuracy and replicability. We utilized this calibration method by referencing the reflection coefficient at 0 V to obtain more accurate test results.

The difference from [17] lies in the placement of the two horn antennas on one side of the surface, with their directions arranged so that they intersect the surface along their extension lines. Subsequently, the measurement system is calibrated using a non-guided through mode for calibration [20]. In the through mode, the Vector Network Analyzer (VNA) measurement system is directly connected to the test ports without passing through any additional components or connectors, which significantly reduces the influence of the environment. We measured the reflection coefficient ($S_{11}$) of the modulating surface, using the increased bias voltage of the modulating surface as the baseline for through calibration at 0 V. The measurement of $S_{11}$ represents the ratio of transmitted to received signals in the two-port network. By measuring $S_{11}$ at different voltages and comparing them with the known 0 V baseline, the reflection coefficients of the two horn antennas are obtained relative to 0 V for each tuning voltage. This process helps in understanding the impact of the modulating surface on signal transmission. The formula for calculating the maximum change in reflection coefficient, $\Delta \Gamma_{max}$, is as follows:

(3) $$\begin{array}{c} \Delta \Gamma_{max}=\Gamma_{max}-\Gamma_{0V}. \end{array}$$

### 4.2. Sample Measurement Results

As illustrated in Figure 5a, using this approach we placed the AFSS screen and horn antennas, with the extension lines of the two horn antennas intersecting the surface. By setting the AFSS bias voltage to 0 V and calibrating the measurement system using the vector network analyzer through mode, the obtained $S_{11}$ represents the magnitude of the transmitted and received signals in the dual-port network.

**Figure 6:**
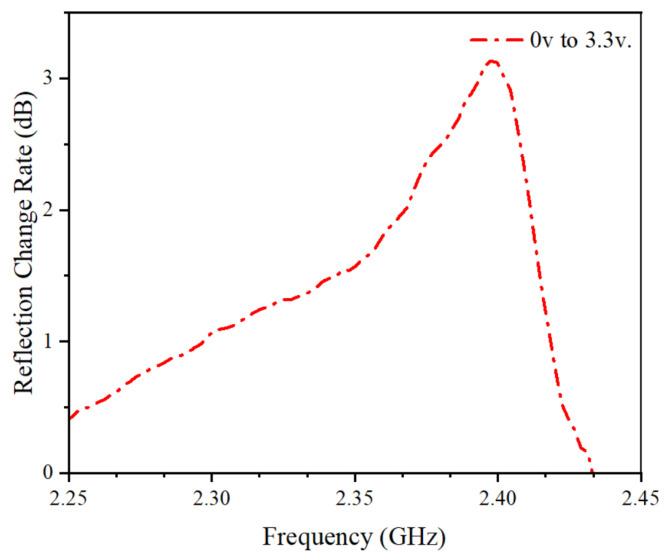
Measurement of 0 −3.3 V reflection change rate at 2.4 GHz environment.

As shown in Figure 6, adjusting the voltage to 3.3 V yields the reflection coefficient of the dual-port. In this study, we focus on the modulation capability at 2.4 GHz. It is evident from the graph that the power signal increases by more than 3 dB at the 2.4 GHz single-frequency point. The miniaturized AFSS designed in this paper achieves signal enhancement for the 2.4 GHz single-frequency point within the range of 0–3.3 V, catering to the bandwidth requirements of IoT systems.

### 4.3. Performance Comparison

As shown in Table 1, the design detailed in this text emphasizes a significantly smaller unit size and lower power consumption compared to the typical designs referenced. At the target frequency, miniaturization of the AFSS inevitably reduces the inductance ($L_{T}$) and increases the operational frequency. This challenge is addressed by the proposed zigzag structure, which enhances mutual inductance (ΔL) between elements under HF signals while also reducing the number of elements in the AFSS array. The unit size of the AFSS is substantially smaller (5.5×5.6 mm${}^{2}$) compared to the sizes in the referenced designs (ranging from 10×10 mm${}^{2}$ to 17.5×17.5 mm${}^{2}$). The design operates at a significantly lower voltage (0–3.3 V) and demonstrates an extraordinarily low total power consumption of 435.6 nW, which is orders of magnitude lower than the power consumption of the referenced designs.

## 5. Conclusions

This study proposes a miniaturized AFSS that is easily integrated into IoT systems. In practical IoT scenarios, the voltage range is typically 0–3.3 V, and the 2.4 GHz frequency is a shared spectrum resource among numerous devices. However, the tunable range of the varactor is relatively small within this voltage range. In such cases, once the main structure is determined, it is desired to maximize the mutual inductance between components by designing a serrated unit. We simulate and illustrate the surface current in the resonant state to demonstrate this idea. The simulation results indicate that the center frequency of the stopband can be tuned within the range of 2.26–2.5 GHz. Subsequently, a prototype of this design was fabricated. Measurement results showed that the reflection efficiency exceeded 3 dB at the 2.4 GHz frequency point when the bias voltage ranged from 0 to 3.3 V, meeting the communication requirements of 2.4 GHz WiFi and making it suitable for IoT low-power devices.

## Figures and Tables

**Table 1:** Comparison of this work with existing articles.

Ref.	Unit Size	AFSS Array	Frequency (GHz)	Voltage (V)	Total Power Consumption
[21]	12.5 × 12.2 mm${}^{2}$	16 × 16	2.92 to 5.74	4–18	1.0368 mW
[22]	17.5 × 17.5 mm${}^{2}$	16 × 16	4.56 to 5.16	0–20	0.77 mW
[23]	10 × 10 mm${}^{2}$	17 × 17	3.7 to 4.27	0–20	1.17045 mW
This paper	5.5 × 5.6 mm${}^{2}$	6 × 10	2.27 to 2.5	0–3.3	237.6 nW

## Data Availability

The original contributions presented in the study are included in the article, further inquiries can be directed to the corresponding authors.
